# Legionella and Air Transport: A Study of Environmental Contamination

**DOI:** 10.3390/ijerph19138069

**Published:** 2022-06-30

**Authors:** Michele Treglia, Margherita Pallocci, Giorgio Ricciardi Tenore, Paola Castellani, Fabrizio Pizzuti, Giovanna Bianco, Pierluigi Passalacqua, Lucilla De Luca, Claudia Zanovello, Daniela Mazzuca, Santo Gratteri, Agostino Messineo, Giuseppe Quintavalle, Luigi Tonino Marsella

**Affiliations:** 1 Department of Biomedicine and Prevention, University of Rome “Tor Vergata”, 00133 Rome, Italy; michele.treglia@uniroma2.it (M.T.); pierluigi.passalacqua@students.uniroma2.eu (P.P.); lucilla.deluca.09@students.uniroma2.eu (L.D.L.); claudia.zanovello@students.uniroma2.eu (C.Z.); agostino.messineo@uniroma2.it (A.M.); marsella@uniroma2.it (L.T.M.); 2 APSSP-Prevention, Safety & Health Association, Velletri, 00049 Rome, Italy; giorgio.ricciarditenore@gmail.com (G.R.T.); castellanipaola58@gmail.com (P.C.); fabriziopizzuti@hotmail.com (F.P.); giovannabianco70@gmail.com (G.B.); 3 Department of Surgical Sciences, University “Magna Græcia” of Catanzaro, 88100 Catanzaro, Italy; danielamazzuca7@gmail.com (D.M.); gratteri@unicz.it (S.G.); 4 Fondazione Policlinico “Tor Vergata”, 00133 Rome, Italy; giuseppe.quintavalle@ptvonline.it

**Keywords:** *Legionella pneumophila*, air transport, microbiological risk assessment, environmental contamination, public health, occupational risk

## Abstract

Introduction: There is growing interest in the public health and transport sectors in research into exposure to biological hazards, considering not only the risks arising from inter-human contagion, but also those related to exposure to the flight environment itself. The aim of this paper is to report data from an investigation into the water and air-conditioning systems of commercial aircraft for the presence of *Legionella* contamination, with a total of 645 water samples taken during the period 2007–2021. Methods: The investigation involved 126 aircraft of six different commercial aircraft types: MD80, Airbus A320 F, Embraer 175/190, AIRBUS A330, Boeing 767 and Boeing 777. Water samples were taken from the water systems (toilet taps, galley and boilers). Each sample was preliminarily subjected to an evaluation of the following parameters: temperature, pH and residual chlorine. The ScanVit^®^ Legionella kit was used for bacteria detection and enumeration. Results: Samples were considered positive if the number of colony-forming units/liter (CFU/L) was >100. For the entire observation period, 45% of the investigated aircraft tested positive. Regarding the overall number of samples analyzed, 68.4% (441/645) were below 100 CFU/L, and thus within the limits allowed by the Italian Guidelines. Conclusions: Water system contamination with Legionella in the air transport field is a real public health issue that should not be underestimated given the heavy passenger traffic. Infection should be considered an occupational risk to which crew members are exposed.

## 1. Introduction

According to a report published by the International Air Transport Association (IATA), in recent years, passenger traffic volume has been growing steadily, exceeding 4.3 billion flights in 2018 [1]. It has been estimated that international airports serve, cumulatively, a million travelers per day; in 2006, 4.4 billion passengers deplaned and enplaned from airports around the world. Long-term forecasts based on traffic statistics predict that this number will double to exceed nine billion passengers per year by 2025 [2]. In light of the above-mentioned volumes, health-related airborne biohazards represent a growing concern in the transport industry due to the serious implications that existing policies, engineering, manufacturing and maintenance protocols concerning these infrastructures may have in the transmission of airborne infectious agents, especially in the case of overcrowding and imbalance between infrastructures’ capacity and their real usage. The consideration of public health safety concerns in the designing and engineering phase, together with a holistic approach involving all stakeholders throughout the life cycle of transport infrastructures, is crucial to minimize future biohazards in the twenty-first century [3]. The transmission of airborne infectious diseases during air travel has become a growing concern, especially during the COVID-19 pandemic. A review of the literature published by the European Centre for Disease Control (ECDC) suggests that the transmission of TB, influenza, SARS, meningococcal disease, and measles on board airplanes occurs relatively frequently [2]. As for the transmission methods, it was highlighted that airborne transmission is the main route of infection spreading by close contact with contagious subjects, as a result of the high traveler density [4]. Furthermore, a potential pathogen contamination of the aircraft cabin environment may represent a route of infection, even in the absence of human-to-human transmission. With regard to the means of transport-related risks specifically attributable to the aircraft drinking-water supply and transfer chain systems, it was shown that these surfaces may provide a real ecological niche favoring the proliferation of microorganisms such as *Pseudomonas* spp. and *Legionella* spp. [5]. Numerous studies report the presence of *Legionella pneumophila* on board trains [6], ferries, cruise ships [7,8,9] and road transport vehicles [10,11]. *Legionella* currently includes 60 species and although half of them are recognized as obligate pathogens for humans, most are considered as facultative pathogens [12]. The best-known and most studied *Legionella* species is *L. pneumophila*, the causative agent for both community-acquired pneumonia known as Legionnaires’ disease and the flu-like illness without pneumonia known as Pontiac Fever. *Non-pneumophila legionella* species usually account for <10% of human infections. Specifically, *L. micdadei*, *L. bozmanii*, *L. longbeachae*, *L. dumoffii*, and *L. feeleii* have been repeatedly isolated in hospitalized patients [13,14]. The natural reservoir of *Legionella* is fresh water, where it grows in biofilms and replicates within other microorganisms [15]. In Europe, Legionnaires’ disease outbreaks are often associated with cooling towers and spas spreading contaminated aerosols. Such outbreaks regularly affect hundreds of people despite strict controls and surveillance measures. Otherwise, most cases are sporadic infections, although contaminated water systems still constitute a severe threat to public health [16]. Despite the well-known fact that *Legionella* can survive and multiply in environments such as water tanks including the water storage tanks of several means of transport, the authors noted the paucity of the existing literature with regard to aircraft systems. To fill this knowledge gap, this paper reports the data from a survey on *L. pneumophila* contamination in the water distribution systems on 126 commercial aircraft, from a total of 645 water samples collected between 2007 and 2021.

## 2. Materials and Methods

**Figure 1:**
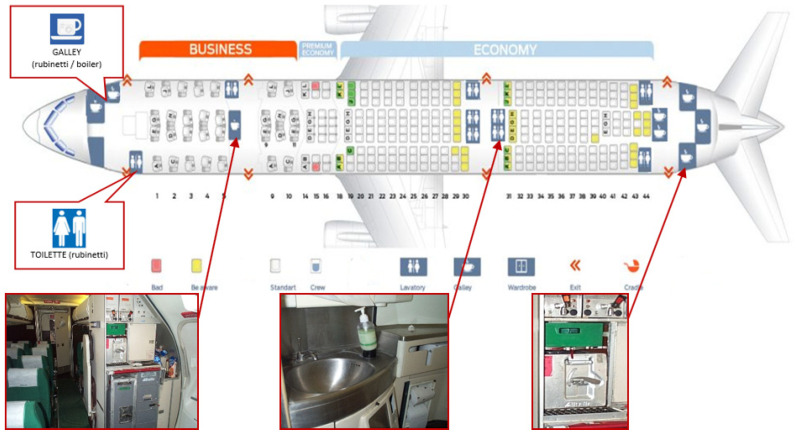
Depiction of the arrangement of water supply points inside an Airbus model aircraft.

This survey was carried out on 126 airliners among 6 different types of commercial aircraft including MD80, Airbus A320 F, Embraer 175/190 for medium-distance flights and Airbus A330, Boeing 767, Boeing 777 for long-distance routes. The water samples were taken from the on-board water systems (toilet taps, galleys and boilers), as proportional to the aircraft configuration (Figure 1). As the first step of the analysis, sampling was performed 30 days after the aircraft water systems’ sanitization, following which sampling was carried out randomly.

**Figure 2:**
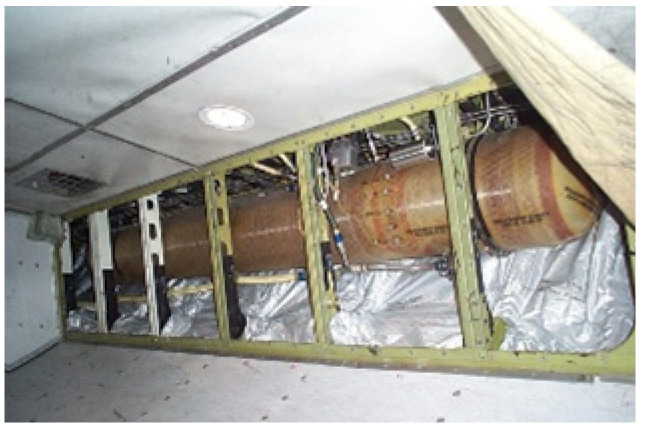
Aircraft potable water tank.

The water is loaded on board the aircraft and usually stored in a specific tank (Figure 2). Water must be potable and in compliance with the international standards adopted by all airports.

**Figure 3:**
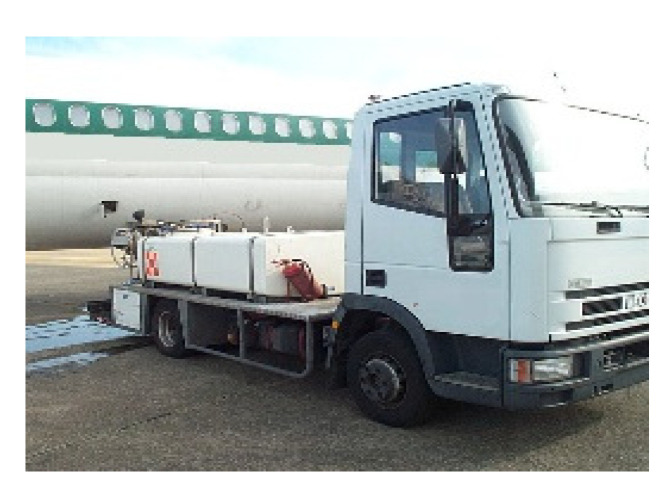
Airport water service trucks.

Stored water is used only for hygiene purposes. For drinking, only bottled mineral water is allowed. Water provided through taps and boilers in a galley is used for the preparation of hot drinks, after heating to about 90 °C (a lethal temperature for *Legionella* bacteria whose inactivation occurs at about 60 °C). *Legionella* is a ubiquitous microorganism, to be monitored both in airport water system pipes and water service trucks (Figure 3) supplying fresh water on board.

The latter undergo monthly disinfection and testing to detect *Legionella* and other microbiological parameters set out in the Italian rule (Legislative Decree No. 31 of 2 February 2001-Implementation of Directive 98/83/EC on the quality of water intended for human consumption) [17].

In all aircraft, drinking water sampling (with determination of temperature, pH, and residual chlorine) was conducted through microbiological analysis to ensure it met the quality standards for water intended for human consumption (Legislative Decree 31/2001). Both sampling and storage procedures were carried out in compliance with the Guidelines for the Prevention and Control of Legionnaires’ disease (State-Region Conference of 7 May 2015: Agreement between the Government, the Regions and the Autonomous Provinces of Trento and Bolzano on the document entitled “Guidelines for the prevention and control of Legionellosis”) [18]. In particular, the temperature of the water sample collected in test sample containers sterilized with a 0.01% solution of sodium thiosulfate was immediately measured with a digital thermometer (Checktemp, Hanna Instruments, Model Hanna Hallo 98501) and the results recorded in the sampling report. No flaming or pre-flushing were involved in the sampling that was carried out using a direct method in order to simulate ordinary usage conditions.

The analytical findings were calculated using the ScanVit^®^ Legionella kit (Vermicon, AG, Munich, Germany), a quantitative test allowing the detection of pathogenic germs in just 3 days instead of the 10–15 days using the standard culture method [19]. Fluorescent-labelled DNA probes are specifically designed to detect *Legionella* spp. and *L. pneumophila* bacteria. During the analysis, these probes penetrate bacterial cells binding to specific target sequences. A light source emitting a specific wavelength of light excites *Legionella pneumophila* bacteria that become colored and visible under the fluorescence microscope (OPTIKA B-353LD). The water sample was kept away from light in a fridge for no more than 24 h and was then filtered (50 mL) by adding an acid buffer into a “funnel” where the filter membrane had been inserted. After filtration, the membrane was placed on one Petri plate of GVPC MEDIUM (Becton Dickinson) agar and incubated at 37 °C in a jar for 72 h in a humid environment and with an enriched CO_2_ atmosphere. After this time, the ScanVit^®^ analysis phase began (2–3 h) performing directly on the membrane filter. Finally, the membrane was placed onto a slide to detect and count the colonies under the fluorescence microscope. The number of colonies x dilution factor produced results measurable in CFU/L. The limit of detection of the method used was 20 CFU/L; however, a sample was considered positive if it exceeded the >100 CFU/L limit as indicated by the reference guidelines. In the range ≤ 100 CFU/L, water samples with results of 0, 20, 40, 60, 80, or 100 were grouped together and considered as negative.

## 3. Results

All on-board aircraft maintenance works depend on the manufacturer’s recommendations in compliance with national and international aviation regulations and technical specifications for the materials and systems involved. Servicing and maintenance aim to ensure that the cleaning of the aircraft water tanks and fresh water supply system are regularly carried out as per the MPD (Aircraft Maintenance Manual) of each aircraft model. All aircraft undergo water system sanitization, according to time ranges, procedures and specifications provided by the manufacturers and by the aviation standards that vary depending on the type of aircraft (Table 1).

**Figure 4:**
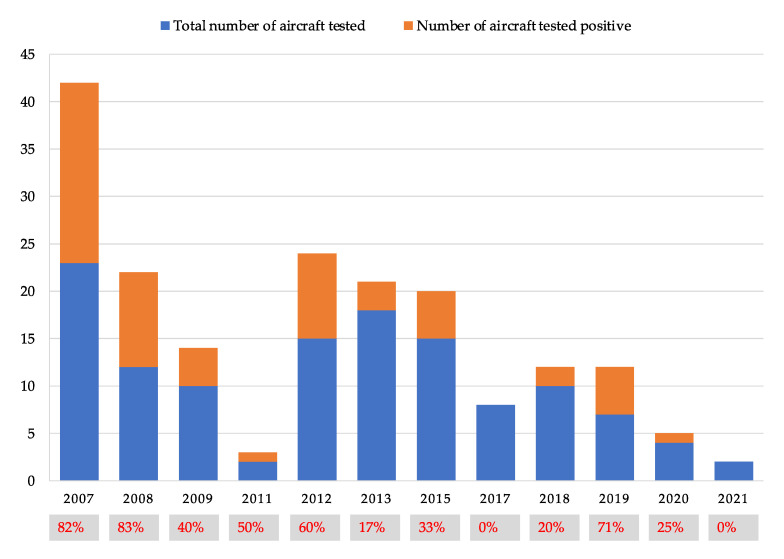
The number of aircraft tested and the number of those found positive—divided by survey year. The percentages of positivity found for each year of investigation are also shown.

Each airline company may set out its own regulations both in terms of the integration and/or variation of the existing manufacturer recommendations by introducing more restrictive rules, and also on the basis of health provisions or regulations, if any, and/or of its own risk prevention measures. At the start of the study (2007), about 20% of the aircraft of the fleet were examined. Subsequently, the number averaged 10%. Air traffic volume declined in 2020 and 2021 due to the COVID-19 pandemic, with a consequent reduction in flights, especially in the case of long-haul routes (Figure 4).

**Figure 5:**
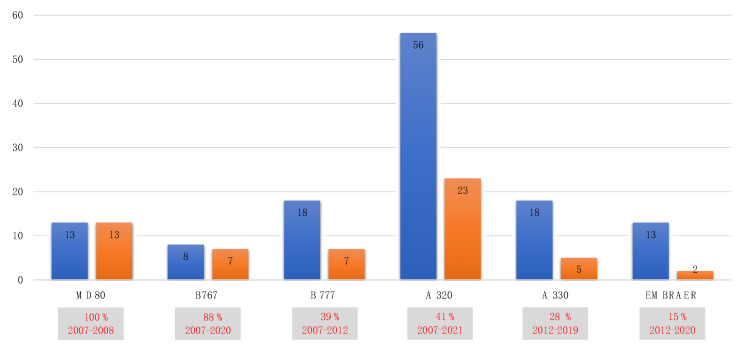
Number of examined airplanes (blue) and number of *Legionella-* positive airplanes (orange) for the entire period of examination.

The average positivity rate for the entire period was 45%. Figure 5 shows the total number of aircraft that tested positive (positivity is always for *Legionella* > 100 CFU/L).

Table 2 shows the number of samples taken for each type of aircraft grouped according to four ranges of results (samples are considered positive if >100 CFU/L).

## 4. Discussion

The results obtained for the long-haul fleet confirmed compliance with maintenance protocols as well as the proper regular sanitization procedures as a prevention against the risk of *Legionella* outbreaks.

It should be noted that the method of analysis used in our study was found to be reliable in specifically detecting *Legionella*. Indeed, it has been shown that acid treatment in the filtration phase and incubation in a selective medium inhibit the growth of other bacterial microorganisms. In a recently published study, the ScanVit^®^ method was compared with the standard method, concluding in favor of the ScanVit^®^ method in the detection of *Legionella* in contaminated water samples [19]. It emerged that the samples > 100 ≤ 1000 CFU in almost all cases had values < 400 CFU/L as a result of the important role of ageing aircraft (e.g., B767). In fact, since 2012 no substantial increase in *Legionella* concentration has been detected: in 68.4% (441/645) of the analyzed samples, *Legionella* remained down by nearly 100 CFU/L and was therefore within the limits allowed under the Italian Guidelines. Only in 7.6% of cases (49/645) were there critical high positivity levels requiring an immediate decontamination. The borderline contamination (100 < CFU/L ≤ 1000) found in 24% of cases (155/645) confirms the need for strict monitoring of the phenomenon.

The reported data show a divergence in the detected positivity, varying according to the type of aircraft, requiring further consideration of their technical features; this is a particularly relevant aspect in terms of prevention. The MD80 aircraft is a low-wing twin-engine tail-thruster passenger aircraft used for short- and medium-distance routes, and was manufactured in the late seventies of the last century. At the beginning of the 2000s, this type of aircraft was gradually retired and replaced with the Airbus A320 This aircraft was fully retired by October 2012. The data refer to the checks performed up to 2008, the year of their retirement from the fleet. The MD80 was the aircraft with the most significant *Legionella* positivity concentrations: 100% of the examined aircraft and 55% of the collected water samples, of which 5% had values > 10,000 CFU/L. These findings are attributable to the age of the aircraft and to a higher concentration of limescale deposits, which are difficult to remove despite regular limescale treatments. Where positive results were found, a full removal and cleaning of the water system was performed after removing the limestone from all the taps and descaling the inside of the boilers. 

The A320 Family is a type of short- and medium-haul aircraft, and has been manufactured since 1987. In 2007, besides other structural changes, a new air filtration system equipped with catalytic converters removing foul odors was installed. This type of aircraft underwent the highest number of checks (44%), being the most highly represented sample in the fleet. The water samples with the highest bacterial density were from the early years of monitoring when the selection and validation phase concerning the disinfection procedures was still in progress.

Among all the examined aircraft, the Embraer is the one with the smallest water system with few water supply points, which is the reason why it was the model with the lowest rate of *Legionella*-positivity. It should also be considered that, upstream of the taps in the galley, there are filters for the removal of odors, flavors, organic pollutants, algae and particles ranging from 2 to 5 microns in diameter, preventing biofilm formation. This is probably compatible with the maximum *Legionella* concentration, <100 CFU/L.

The B767 is the most used airliner for transatlantic flights from North America to Europe that is still being produced. The checks were carried out until 2012 when it was retired from the fleet. The B777 is a more technologically advanced, wide-body commercial airliner intended for long-distance routes. It is equipped with the most complex water system with the highest number of water supply points among all the aircraft under examination and consequently with the second largest number of collected samples (on average nine per airliner). With regard to these two types of aircraft, the results were substantially similar.

Finally, the A330 is a twin-engine airliner designed in the late 1980s and used for medium- and long-distance flights. The checks started in 2013, the year of its inclusion in the examined fleet. The best results were obtained for this aircraft as none of the analyzed samples showed a positivity > 1000 CFU/L, a result that can be probably justified by the shorter periodic disinfection interval of the water system.

Although in the existing literature no *Legionella* disease-related report following air travel has been found, the results of this study show that the risk of infection is not zero.

As a general consideration, it is known that the risk of developing the disease following exposure is related to individual susceptibility, the intensity of exposure assessed in terms of infectious charge, and exposure time. Therefore, it is clear that the infectious threat could involve not only passengers, but also the flight crew, for whom it represents a potential occupational risk.

On the other hand, additional studies have investigated environmental contamination by other microorganisms. A study investigating the microbiological quality of water in a sample of short- and long-haul aircraft showed poorer water quality in the latter, suggesting the need for better sanitization protocols. In detail, the authors documented the presence of 37 different genera, of which the most represented species were, among the Gram-positive species, *Bacilli* and *Acinetobacter*, and among Gram-negative species, *γ, β, α- Proteobacteria, Flavobacteria*, *Sphingobacteria* and *Cytophaga* (in the analysis performed, however, no microorganisms capable of causing serious diseases were found, such as the Shiga toxin, producing *E. coli*, *Enterococcus* and *Legionella*) [20].

A study conducted in order to characterize the microbiome on contact surfaces (toilet seats and doors) in aircraft cabins revealed the prevalence of cutaneous commensal microorganisms belonging to the *Propionibacteriaceae* family and other microorganisms of bacterial origin (*Enterobacteriaceae*, *Staphylococcaceae*, *Streptococcaceae*, *Corynebacteriaceae* and *Burkholderiaceae*) [21]. In particular, the surfaces of lavatories and relevant areas were one of the major sources of contamination. An investigation based on a PCR (Polymerase Chain Reaction) approach documented the presence of a broad spectrum of bacterial contamination (58 genera) on the surfaces analyzed, including toilet seats, floors, sink handles and sink faucets. The most frequently identified microorganisms belonged to *Streptococci*, *Staphylococci*, *Corynebacteria*, *Propionibacteri* and *Kocuria*. In the samples taken from the toilet floor in particular, the preponderant presence of *Corynebacteria* compared to the other surfaces was evident, whereas in the toilet seat, *streptococci* represented the majority [22]. Other authors investigating the contamination of other surfaces such as those of seats, in particular armrests, and tray tables have shown the presence of bacteria belonging to the skin flora (mainly Gram-positive cocci) and fungi (although the latter at lower concentrations than bacterial microorganisms) [23].

The infectious dose for humans as well as the factors triggering a different virulence in several *Legionella* species and serogroups is still unknown. They may depend on the hydrophobic nature of the surface, the survival of *Legionella* in aerosols, or its ability to grow in environmental amoebae. Even the *Legionella* physiological status triggering the infection remains unknown, although it may include both the stationary growth phase and the log phase, as well as the so-called spore-like forms. Legionellosis is normally achieved via the respiratory route through the inhalation, aspiration or microaspiration of aerosols containing *Legionella*, or particles derived by drying. Droplets may originate by spraying water, by bubbling air into it, or by impact on solid surfaces. The danger of these water particles is inversely proportional to their size. Droplets smaller than 5μ in diameter reach the lower respiratory tract more easily. In this regard, it is emphasized that in on-board toilets, the water from lavatories is not supplied under pressure, a factor reducing the infectious risk.

A risk assessment may be obtained using the quantitative microbial risk assessment (QMRA). This approach, introduced for the first time in the 1990s, allows a quantitative analysis of the risk in terms of infection, disease, or mortality from microbial pathogens. This method was used in order to assess the risk of pneumonia associated with exposure to aerosols from hot tubs and spas contaminated by *Legionella* and, recently, also in the context of long-distance public transport (LDT). It was reported that approximately 55% of the water samples were positive for *L. pneumophila*, and the most frequently isolated was *L. pneumophila* sg1. Subsequently, a sink-specific aerosolization ratio was applied to calculate the inhaled dose, also considering the inhalation rate and exposure time, which were used as stochastic parameters based on literature data. At *L. pneumophila* concentrations ≤100 CFU/L, the health risk was approximately 1 infection per 1 million exposures, increasing to 5 infections per 10,000 exposures when concentrations were ≥10,000 CFU/L. This study demonstrated a low risk of *Legionella* infection from taps on LDT even though LDT may be used by people who are highly susceptible to the development of a severe form of the disease, due to their immunological status or other predisposing factors [5].

### Limitations of the Study

In this study, analysis was limited to samples collected from the on-board toilets and cooking areas (galley and boiler). Contamination by the wastewater toilets could potentially have produced contaminated aerosols. It should also be noted that the performed study assessed the presence of microorganisms on board aircraft without considering other potential contamination sources such as the tankers used for the water supply.

## 5. Conclusions

This study investigated the environmental contamination with *Legionella* on commercial aircraft, an aspect poorly discussed in the literature. Water system contamination with *Legionella* in the air transport field is a real public health issue that should not be underestimated given the heavy passenger traffic and the fact that passengers include debilitated people, children, the elderly, and individuals more susceptible to *Legionella* infections. It is also necessary to consider the potential implications in terms of occupational risk, a context that deserves particular attention, especially for prevention. In 24% of cases (155/645), borderline contamination (over 100 but less than 1000 CFU/L) was found, indicating the need for careful surveillance of the phenomenon. As research has shown, keeping *Legionella* under control in a water distribution system always requires an optimal systematic schedule of disinfection, water management, nutrient limitation, and temperature control [16,24]. In this regard, it should be noted that the decrease in the positivity rate observed in our study is attributable to the effectiveness of the sanitization protocols put in place. However, it is possible to suggest that the interval between checks should be reduced, in order to further improve and implement safety on board aircraft.

## Figures and Tables

**Table 1:** Water supply points according to aircraft type and frequency and sanitization methods applied in the investigated aircraft compared to those recommended by the aircraft manufacturer.

	Embraer 175/190			MD80	Airbus A320F		Airbus A330	Boeing B767		Boeing B777		
Tank capacity (in liters)	60			~200	200		450	~450		3 × 454 (1 per potable water)		
Water supply points	Toilets (2)	Galley (2)		Toilets (3)	Galley (2)		Toilette (8)	Galley (3)		Toilette (10)	Galley (3)	
	Taps 2	Taps 2	Boiler 2	Taps 3	Taps 2	Boiler 2	Taps 8	Taps 2	Boiler 4	Taps 10	Taps 3	Boiler 6
	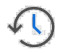 Manufacturer sanitization frequency recommendations				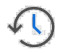 Airline company sanitization frequency recommendations			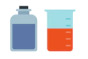 Products for sanitization				
Embraer 175/190	15 months				~250 dd			70% calcium hypochlorite solution or 10% sodium hypochlorite solution				
MD80 Airbus A320F	1500 flight hours				~250 dd			50 ppm 13% sodium hypochlorite solution at or 100 ppm free Cl				
Airbus A330	3 months				~90 dd			Calcium hypochlorite solution sodium hypochlorite 50% hydrogen peroxide (H_2_O_2_)				
Boeing B767 Boeing B777	150 dd or 2000 flight hours				~130 dd			Chlorine dioxide or Chloramine				

**Table 2:** Number of samples per type of aircraft and related results.

Results (CFU/L)	MD80	B767	B777	A320F	A330	Embraer	All Types
≤100	29 (45%)	70 (67%)	125 (77%)	139 (64%)	49 (78%)	29 (91%)	441 (68.4%)
>100 ≤1000	16 (25%)	31 (29%)	36 (22%)	56 (25%)	14 (22%)	2 (6%)	155 (24%)
>1000 ≤10,000	16 (25%)	4 (4%)	2 (1%)	22 (10%)	0	1 (3%)	45 (7%)
>10,000	3 (5%)	0	0	1 (0,5%)	0	0	4 (0.6%)
Total Samples	64	105	163	218	63	32	645

## Data Availability

Data available on request due to privacy restrictions. The data presented in this study are available on request from the corresponding author.
